# Time to positivity in blood cultures of adults with S*treptococcus pneumoniae *bacteremia

**DOI:** 10.1186/1471-2334-6-79

**Published:** 2006-04-27

**Authors:** Galo Peralta, María José Rodríguez-Lera, Jose Carlos Garrido, Luis Ansorena, María Pía Roiz

**Affiliations:** 1 Internal Medicine Service, Sierrallana Hospital, Barrio de Ganzo s/n, 39120 Torrelavega, Cantabria, Spain; 2 Emergency Service, Sierrallana Hospital, Barrio de Ganzo s/n, 39120 Torrelavega, Cantabria, Spain; 3 Laboratory Service, Sierrallana Hospital, Barrio de Ganzo s/n, 39120 Torrelavega, Cantabria, Spain; 4 Admission Service, Sierrallana Hospital, Barrio de Ganzo s/n, 39120 Torrelavega, Cantabria, Spain; 5 Microbiology Service, Sierrallana Hospital, Barrio de Ganzo s/n, 39120 Torrelavega, Cantabria, Spain

## Abstract

**Background:**

*previous* studies have established that bacterial blood concentration is related with clinical outcome. Time to positivity of blood cultures (TTP) has relationship with bacterial blood concentration and could be related with prognosis. As there is scarce information about the usefulness of TTP, we study the relationship of TTP with clinical parameters in patients with *Streptococcus pneumoniae *bacteremia.

**Methods:**

TTP of all cases of *Streptococcus pneumoniae *bacteremia, detected between January 1995 and December 2004 using the BacT/Alert automated blood culture system in a teaching community hospital was analyzed. When multiple cultures were positive only the shortest TTP was selected for the analysis.

**Results:**

*in* the study period 105 patients with *Streptococcus pneumoniae *bacteremia were detected. Median TTP was 14.1 hours (range 1.2 h to 127 h). Immunosuppressed patients (n = 5), patients with confusion (n = 19), severe sepsis or shock at the time of blood culture extraction (n = 12), those with a diagnosis of meningitis (n = 7) and those admitted to the ICU (n = 14) had lower TTP. Patients with TTP in the first quartile were more frequently hospitalized, admitted to the ICU, had meningitis, a non-pneumonic origin of the bacteremia, and a higher number of positive blood cultures than patients with TTP in the fourth quartile. None of the patients with TTP in the 90^th ^decile had any of these factors associated with shorter TTP, and eight out of ten patients with TTP in the 10^th ^decile had at least one of these factors. The number of positive blood cultures had an inverse correlation with TTP, suggesting a relationship of TTP with bacterial blood concentration.

**Conclusion:**

Our data support the relationship of TTP with several clinical parameters in patients with *Streptococcus pneumoniae *bacteremia, and its potential usefulness as a surrogate marker of outcome.

## Background

*Streptococcus pneumoniae *is a major cause of pneumonia, meningitis and bacteremia and is among the leading infectious causes of illness and death worldwide [1]. The mortality of pneumococcal bacteremia remains high and is related to several prognostic factors [2-7]. The time to positivity of blood cultures (TTP) of patients with *Streptococcus pneumoniae *bacteremia has previously been explored as a marker of outcome, but no relationship with clinical or laboratory parameters has been found in children [8]. TTP of simulated blood cultures with Staphylococcus epidermidis has a linear correlation with initial bacterial concentration [9,10] although this has not studied with *Streptococcus pneumoniae*. In addition, several researchers have found a correlation between concentration of bacteria in blood and the presence of meningitis and its prognosis [11-16]. Because it is unknown if TTP correlates with outcome we decide to explore its relationship with clinical parameters and prognosis in adult patients with *Streptococcus pneumoniae *bacteremia.

## Methods

### Patients

The study was conducted at the Sierrallana Hospital, a 220-bed community hospital. All the blood cultures taken from January 1995 to December 2004 were reviewed and patients whose blood cultures yielded *Streptococcus pneumoniae *were selected for the study.

A standardized data collection form was used to review the hospital records of patients with pneumococcal bacteremia. Pneumococcal pneumonia was defined by the presence of acute respiratory symptoms and fever in association with new radiographic infiltrates in a patient with pneumococcal bacteremia. Pneumococcal meningitis was defined by compatible clinical symptoms including headache and meningismus, and growth of *Streptococcus pneumoniae *or pleocytosis of at least 100 neutrophils per cubic millimeter, in cerebrospinal fluid. Primary bloodstream infection was defined by the documentation of pneumococcal bacteremia in the absence of any recognized local infection, which might have given rise to the bacteremia. Sepsis, severe sepsis, and septic shock were defined according to American College of Chest Physicians and Society of Critical Care Medicine consensus statement [17]. The study has been approved by the local ethics committee.

### Blood cultures

The recommended practice in our hospital is to obtain 20 mL of venous blood and innoculate it in equal parts into one aerobic (BacT/ALERT FA aerobic, bioMerieux Corporation, Durham, North Carolina) and one anaerobic blood culture bottle (BacT/ALERT FN, bioMerieux). Blood extraction is usually performed three times at intervals of 30 minutes. The blood culture bottles obtained for each patient are immediately sent to the microbiology laboratory and introduced into the blood culture instrument (BacT/ALERT microbial detection system, bioMerieux), which has a colorimetric carbon dioxide sensor to measure microbial growth. The system tests for carbon dioxide production and records the time elapsed from the placement of each blood culture bottle in the system to the detection of microbial growth. This time is considered as the TTP. The bottles are incubated for five days. When multiple cultures were positive only the shortest TTP was selected for the analysis. *Streptococcus pneumoniae *were identified using standard procedures. Time interval between obtaining the blood for culture and incubation of the bottles was not recorded.

### Statistical analysis

Otherwise indicated data are expressed as mean ± standard deviation. In statistical analyses, Student's t test (two tailed) and the Mann-Whitney rank-sum test were used for the comparison of mean values, Fisher's exact test and the χ^2 ^test were used for the assessment of proportions, and Sperman's correlation coefficient to explore the correlation between two variables. The statistical analysis was performed using SPSS software, version 12.0.

## Results

### Patients

One hundred and five patients with *Streptococcus pneumoniae *blood isolates were identified at the Sierrallana Hospital between January 1, 1995, and December 31, 2004. The overall mean age was 65.9 years (range: 16–93 years). Forty four patients (41.9%) were female. Ninety four patients (89.5%) were admitted to hospital and positive blood cultures were obtained from the remaining 11 patients (10.5%) at emergency visits, but they were not hospitalized. The mean hospital stay was 13.9 days (range: 1–54 days).

Concurrent pneumonia was present in 85 patients (81%), meningitis in seven (6.7%), and sinusitis, spontaneous bacterial peritonitis, cholangitis, and hip prosthesis infection, in one patient each. Endocarditis was also diagnosed in one patient with meningitis. No source of bacteremia was identified in 8 patients (7.6%). The origin of the bacteremia was nosocomial in only two cases. Eleven patients (10.5%) had severe sepsis and one patient met septic shock criteria.

Nine patients (8.6%) were on treatment with antibiotics at the moment of the blood culture extraction. Three with oral macrolides, one with an oral and one with a intramuscular cephalosporin, one with oral ciprofloxacin, one with oral ampicillin-sulbactam, and one with a non specified oral antibiotic.

Five patients had immunosuppression (4.8%), three due to steroid treatment, one due to HIV infection, and one due to an advanced multiple myeloma. Fourteen patients (13.3%) were admitted to the ICU, six because of meningitis, five due to respiratory insufficiency requiring mechanical ventilation, one due to multiorgan failure, one due to digestive haemorrhage, and one due to a supraventricular tachycardia. Of the 13 patients who died, five had bilateral pneumonia, three had severe sepsis and one had septic shock. The cause of death was respiratory failure in ten patients, cardiac failure in two and septic shock in one. Only one of the patients who died had been admitted to the ICU.

### Blood cultures

The number of the blood culture bottles obtained from each patient with pneumococcal bacteremia was: six bottles in 97 patients (92.4%), five bottles in one patient, four bottles in two patients, three bottles in one patient, and two bottles in four patients. In one patient whose bacteremia had no apparent origin, Escherichia coli was isolated together with *Streptococcus pneumoniae *from one blood culture, and its TTP was not considered for the analysis. In another two of the six bottles obtained from this patient, *Streptococcus pneumoniae *grew alone and the TTP was included in the analysis.

Three *Streptococcus pneumoniae *isolated from blood cultures had a high-level of penicillin resistance (MIC ≥ 2 μg/mL) and 17 had intermediate penicillin resistance (MIC between 0.1 and 2 μg/mL).

### Time to positivity

TTP was available from all patients. All but two patients had a TTP shorter than 22 hours. The two other patients had TTPs of 120 hours and 127 hours. Pneumonia was diagnosed in both patients, who had non-severe sepsis and survived without ICU support.

**Figure 1:**
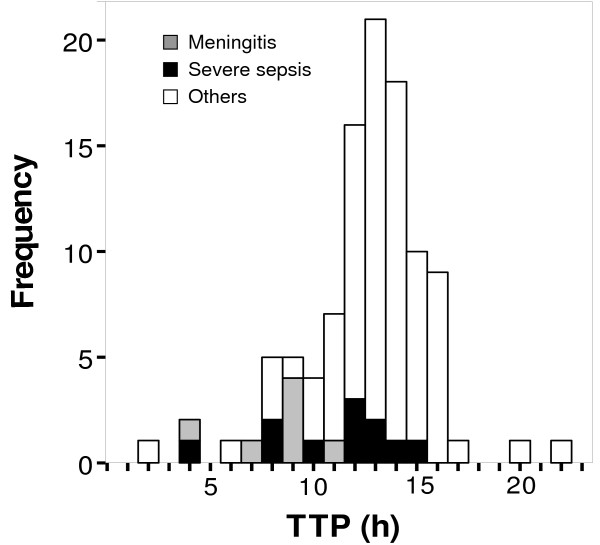
TTP in the 105 patients with *Streptococcus pneumoniae *bacteremia. Two patients with extreme values of TTP are excluded (120 h and 122 h respectively). One patient in the group of meningitis had also severe sepsis.

**Figure 2:**
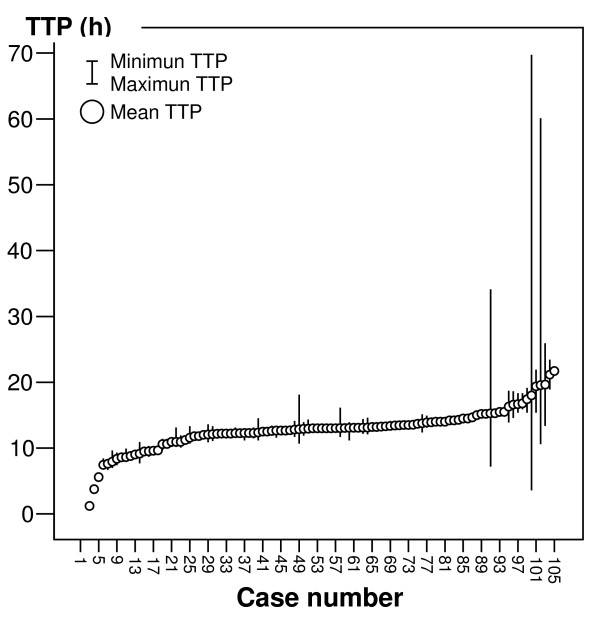
The minimal, maximal and mean TTP value of the blood cultures of each patient are represented. Two patients with extreme values of TTP are excluded (120 h and 122 h respectively, each only with one positive blood culture).

Median TTP was 14.1 h (range: 1.2 to 127 h) (Figure 1). The 25^th ^percentile of TTP was 10.9 h, the 75^th ^percentile was 13.7 h, and the 10^th ^and 90^th ^deciles of TTP were 7.9 h and 15.4 h respectively. Among 78 patients with more than one positive blood culture median maximal difference of the TTP among the blood culture bottles of each patient was 1.1 h (interquartile range 0.67–2.4 h) and the median intrapatient TTP variation coefficient was 4.2 (interquartile range 1.9–22.9) (Figure 2). Among 75 patients with aerobes and anaerobes positive blood cultures, in 40 (53.3%) the anaerobes bottles had lower TTP. The median difference among shortest aerobe bottles TTP and anaerobe bottles TTP of each of these patients was -0.08 h (interquartile range -0.57–0.33 h).

**Figure 3:**
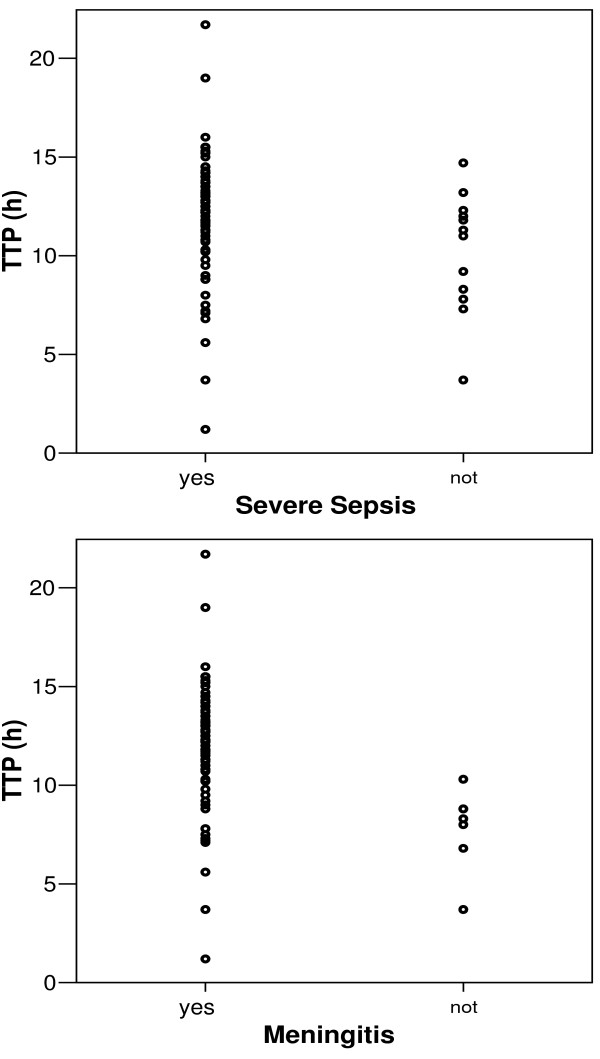
Comparative representation of TTP in patients with and without severe sepsis and meningitis.

No statistically significant differences were detected in TPP depending on sex, age, comorbidities, existence of chills, prior antibiotic treatment, bilateral pneumonia, *Streptococcus pneumoniae *penicillin susceptibility or fatal outcome. Immunosuppressed patients, those with confusion, severe sepsis or shock at the time of blood culture extraction had statistically significant lower TTP, as well as those diagnosed with meningitis and those admitted to the ICU (Table 1, Figure 3).

**Table 1:** Comparison of TTP values of the 103 patients in relation to clinical parameters.

**Characteristic (n)**	**TTP**		
	
	**With the factor**	**Without the factor**	**p**
**Basal characteristics**			
Male (n = 61)	12.06 ± 3.04	11.85 ± 2.84	>0.1
Older than 75 y (n = 41)	11.81 ± 3.64	12.07 ± 2.44	>0.1
Hospitalized (n = 94)	11.79 ± 3.06	13.42 ± 1.08	0.001
Previous antibiotic* (n = 9)	11.3 ± 3.84	12.03 ± 2.87	>0.1
Penicillin susceptible (n = 85)	11.77 ± 2.88	12.8 ± 3.19	>0.1
			
**Comorbidities**			
Alcoholism (n = 17)	12.92 ± 2.48	11.78 ± 3.01	>0.1
COPD (n = 31)	12.61 ± 3.74	11.72 ± 2.56	>0.07
Diabetes (n = 11)	11.77 ± 1.43	11.99 ± 3.07	>0.1
Cirrhosis (n = 7)	11.42 ± 2.63	12 ± 2.98	>0.1
Immunosuppression (n = 5)	9.4 ± 5.2	12.1 ± 2.77	0.05
Dementia (n = 6)	11.63 ± 4.4	11.99 ± 2.89	>0.1
			
**Clinical presentation**			
Confusion (n = 19)	10.36 ± 2.58	12.33 ± 2.68	0.01
Severe sepsis or shock (n = 12)	10.22 ± 3.05	12.2 ± 2.87	0.02
Chills (n = 34)	12.17 ± 2.59	11.88 ± 3.11	>0.1
Bilateral pneumonia (n = 13)	12.24 ± 3.89	11.98 ± 2.80	>0.1
			
**Source of bacteremia**			
Meningitis (n = 7)	7.81 ± 2.1	12.27 ± 2.78	<0.001
Pneumonia (n = 85)	12.19 ± 2.71	11.05 ± 3.72	<0.03
Primary (n = 8)	13.33 ± 3.55	11.85 ± 2.89	>0.1
			
**Outcome**			
ICU (n = 14)	9.22 ± 2.48	12.4 ± 2.79	<0.001
Exitus (n = 13)	11.40 ± 4.17	12.05 ± 2.75	>0.1

* Including ambulatory treatment immediately before blood culture.

When we analyzed the differences between the characteristics of patients with TTP in the first quartile and in the fourth quartile, we detected statistically significant differences between both groups in the proportion of hospitalized patients, number of patients admitted to ICU, and those with meningitis. Patients with TTP in the first quartile also a higher number of positive blood cultures than patients with TTP in the fourth quartile (Table 2).

**Table 2:** Comparison of characteristics of patients with TTP in the 1^st^and 4^th^quartile.

**Characteristic**	**1**^**st**^**TTP quartile**	**2**^**th**^**and 3**^**rd**^**TTP quartiles**	**4**^**th**^**TTP quartile**	p*
**Total (n)**	26	54	25	
**TTP m**edian (interquartile range)	8.8 (7.18–10.2)	12.4 (11.8–13)	23.94 (14.2–15.5)	
**Basal characteristics**				
Age (years)	68.5 ± 18.4	64.6 ± 18.3	67.32 ± 17.62	>0.1
Male n (%)	14 (53.8)	33 (61.1)	14 (56)	>0.1
Time evolution (days)	3.6 ± 2.8	3.4 ± 3.3	3.7 ± 3.4	>0.1
Hospitalized n (%)	26 (100)	46 (88.5)	20 (80)	0.02
Previous antibiotic n (%)	3 (11.5)	4 (7.4)	2 (8)	>0.1
**Comorbidities**				
Alcoholism n (%)	1 (3.8)	11 (20.4)	5 (20)	>0.1
COPD n (%)	6 (23.1)	12 (22.2)	13 (52)	0.03
Diabetes n (%)	2 (7.7)	8 (14.8)	1 (4.0)	>0.1
Neoplasm n (%)	0	3 (5.6)	2 (8)	>0.1
Immunosuppression n (%)	2 (7.7)	2 (3.7)	1 (4)	>0.1
Dementia n (%)	1 (3.8)	3 (5.6)	2 (8)	>0.1
**Clinical presentation**				
T^a^(°C)	38.3 ± 0.7	37.9 ± 1	37.8 ± 0.8	>0.1
Chills n (%)	7 (26.9)	18 (33.3)	9 (36)	>0.1
Confusion n (%)	9 (34.6)	6 (11.1)	4 (16)	0.08
Severe sepsis or shock n (%)	5 (19.2)	6 (11.1)	1 (4)	0.09
Bilateral pneumonia n (%)	3 (11.5)	5 (9.3)	5 (20)	>0.1
**Origin of bacteremia**				
Meningitis n (%)	7 (26.9)	0	0	<0.001
Pneumonia n (%)	17 (65.4)	45 (79.6)	23 (92)	0.02
Others n (%)	2 (7.7)	8 (14.8)	3 (12)	>0.1
**Laboratory parameters**				
Penicillin susceptible n (%)	21 (80.8)	45 (83.3)	19 (76)	>0.1
WBC (cells/μL)	15880 ± 5120	19070 ± 12926	19633 ± 10812	0.01
Neutrophils (%)	89.7 ± 6.06	78.6 ± 15.8	85.4 ± 10.2	0.06
Platelets (cells/μL)	211580 ± 64385	188340 ± 76700	234000 ± 116387	0.03
Creatinine level (mg/dL)	1.3 ± 0.6	1.1 ± 0.5	1.3 ± 1	>0.1
Albumin (mg/dL)	2.8 ± 0.7	3 ± 0.6	3 ± 0.6	>0.1
Cholesterol (mg/dL)	131 ± 56.5	139.6 ± 57.7	177 ± 53.3	>0.1
Positive blood cultures (n)	5 ± 1.56	4 ± 2	2 ± 1.4	<0.001
**Outcome**				
Hospital stay (days)	17.3 ± 12.8	23.64 ± 71.6	11.4 ± 7.9	0.07
Exitus n (%)	4 (15.4)	5 (9.3)	4 (16)	>0.1
ICU n (%)	9 (34.6)	5 (9.3)	0	<0.001

*P values are referred to the comparisons between patients in the 1^st ^and the in 4^th ^quartile TTP

Eight of the ten patients with TTP in the 10^th ^decile had at least a factor associated with shorter TTP: four had severe sepsis (one with multiorgan failure), two were immunosuppressed (one treated with steroids and the other diagnosed with multiple myeloma), two were diagnosed with meningitis (one of them also had an endocarditis) and four required a stay in the ICU. None of the patients with TTP in the 90^th ^decile had meningitis, severe sepsis, shock or immunosuppression, nor required ICU admission.

**Figure 4:**
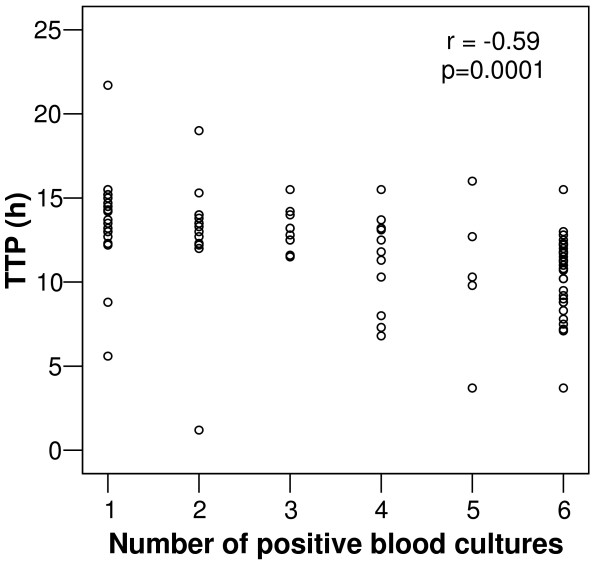
Correlation of the TTP and the number of positive blood cultures of each episode of pneumococcal bacteremia. Two patients with extreme values with only positive one blood culture are excluded (120 h and 122 h respectively).

A significant correlation was found between the TTP and the number of positive blood cultures from each episode (Figure 4).

## Discussion

In our patients with *Streptococcus pneumoniae *bacteremia we found a relationship between TTP and several clinical parameters. TTP is shorter in hospitalized patients, patients with immunosuppression, meningitis, confusion, severe sepsis or shock and in those admitted to ICU. Moreover, most of the patients with the shortest TTP (TTP in the first decile) had a factor that could explain this short TTP. However, a shorter TTP was not detected in the patients who died. The dissociation of TTP between ICU patients and those who died may be attributable to the fact that only one of the patients who died had been admitted to ICU. The other patients with a fatal outcome had not been admitted to ICU because of a calamitous basal condition, which probably contributed to the fatal outcome. On the other hand the first cause of death was bilateral pneumonia, a process that was not associated with a shorter TTP in our patients.

Our study is retrospective and the results must be interpreted cautiously, though they are supported by several previous investigations. In vitro experiments on simulated blood cultures with the new automated blood culture systems have found a relationship between TTP and bacterial [9,10] and *Candida spp*. blood concentration [18]. In children, bacterial blood concentration correlates the prognosis and the appearance of meningitis, not only in cases of pneumococcal bacteremia [11,14] but also in cases of *Haemophilus influence *[11-15] and *Neisseria meningitidis *bacteremia [12]. A correlation also exists between the number of organisms in the CSF and in blood in patients with bacterial meningitis [16]. As TTP is determined by the bacterial blood concentration, it has been suggested as a surrogate marker for the initial bacterial density [10,19,20][21], so it is logical to think that TTP should be in relation to several clinical parameters. In fact TTP is longer when the microorganisms isolated in blood cultures are contaminants in patients with sickle cell disease [22], and in children [23]. A recent study concluded that TTP in patients with *Staphylococcus aureus *bacteremia is an independent predictor of an endovascular source of infection, metastatic infection or extended bacteremia [24].

Recently, it has also been proved that the value of the difference between the TTP of blood cultures drawn through the central venous catheter and the TTP of those drawn from the peripheral vein is highly diagnostic of catheter-related bloodstream infection in patients with long-term catheters [19,20,25]. It has been proposed that this parameter could substitute the differential quantitative cultures used previously in the diagnosis of catheter related infection [19,20,24,25].

The effect of several factors other than bacterial concentration on TTP has been also addressed. The influence of the different automated blood culture systems on TTP is well documented [26-28]. Also, there are great differences in TTP between different microorganisms in the clinical setting [22,26-28]. One factor that can alter TTP is the presence of antibiotic. A recent study has evaluated the effect of various antibiotics on TTP, detecting differences in their prolonging effect depending on the blood culture system used [29]. We have not found differences in TTP among patients treated with antibiotics when blood cultures we performed. Several explanations can be provide for this finding. It is possible that antibiotic treatment was administered earlier to the more seriously ill subgroup of patients. On the other hand the low plasmatic concentrations achieved by a some oral antibiotic treatment could have an negligible effect on TTP. This aspect could be of special relevance in the case of the macrolides in view of the low plasmatic concentrations of this antibiotics [30].

Previous studies focusing on TTP in patients with *Streptococcus pneumoniae *bacteremia have found similar mean TTP values to ours [8,26-29]. However, the only study that analyzed the relationship of TTP with clinical parameters in children with *Streptococcus pneumoniae *did not find any [8]. Several factors may explain these dissimilar results, such as, for example, the study population (paediatric in their study versus adult population in ours), the number of blood cultures per patient (one versus three), the volume of blood per blood culture bottle (one to three millilitres versus ten millilitres) and the blood culture system used (Bactec versus BacT/Alert) [8]. Other factors not controlled, such as an erratic delay in placing blood culture bottles in the automated blood culture system after extraction and the variability in the quantity of the blood drawn inoculated in culture bottles may have contributed to the different results.

Another point of interest in our patients is that TTP also has an inverse relationship with the number of positive blood cultures. As has been said, in simulated blood cultures when bacterial blood concentration increases, TTP decreases [9,10], and when bacterial charge increases, the probability of positive blood cultures also increases [9]. If both TTP and the number of positive blood cultures depend on bacterial concentration, their relationship, can be expected, as we have found. This supports the importance of bacterial concentration as a determinant of TTP in patients.

Our study has several limitations. Due to its retrospective design data are obtained from patients' records and some data could be missed. In addition, time waiting to culture after extraction and volume of blood inoculated in blood cultured bottles could influence the TTP and were not recorded. Other limitation is the low number of patients with some of the analyzed characteristics which impede obtain solid conclusions about the relations of TTP with them. Moreover the population studied had a low mortality and a relatively low incidence of comorbidity so the extrapolation of our data to other different populations with higher mortality and degree of immunosuppression should me made with caution. The influence of the blood culture system used in the TTP values also complicate comparisons among different institutions.

Our data support the relationship of TTP with several clinical parameters in patients with *Streptococcus pneumoniae *bacteremia, although their clinical usefulness probably is limited in view of the narrow spread of their values. However the presence of a low TTP in a patient with *Streptococcus pneumoniae *bacteremia could alert, for example, for the presence of a meningitis or immunosuppression in patients without a severe sepsis. The refinement of TTP data, including the control of potential influencing factors such as time awaiting culture, blood volume per culture bottle, and perhaps prior antibiotic administration could even improve its relationship with clinical parameters in patients with *Streptococcus pneumonia *bacteremia.

## Conclusion

Our data support the relationship of TTP with several clinical parameters in patients with *Streptococcus pneumoniae *bacteremia, and its potential usefulness as a surrogate marker of outcome.

## Competing interests

The author(s) declare that they have no competing interests.

## Authors' contributions

GP conceived the study, participated in the design, the acquisition, analysis and interpretation of data, and performed the statistical analysis.

MJRL participated in the design of the study and its coordination and analysis of data.

JCG participated in the acquisition, analysis and interpretation of data.

LA participated in the acquisition, analysis and interpretation of data.

MPR carried out the microbiological studies, and participated in the coordination, acquisition, analysis and interpretation of data.

## Pre-publication history

The pre-publication history for this paper can be accessed here:


